# Physico-Chemical Properties, Antioxidant Activity and Mineral Contents of Pineapple Genotypes Grown in China

**DOI:** 10.3390/molecules19068518

**Published:** 2014-06-23

**Authors:** Xin-Hua Lu, De-Quan Sun, Qing-Song Wu, Sheng-Hui Liu, Guang-Ming Sun

**Affiliations:** Germplasm Repository of Pineapple (*Ananas comosus*) Zhanjiang City, Ministry of Agriculture, South Subtropical Crop Research Institute, Chinese Academy of Tropical Agricultural Sciences, Zhanjiang, Guangdong 524091, China

**Keywords:** pineapple, genotypes, physico-chemical properties, antioxidant activity, minerals

## Abstract

The fruit physico-chemical properties, antioxidant activity and mineral contents of 26 pineapple [*Ananas comosus* (L.) Merr.] genotypes grown in China were measured. The results showed great quantitative differences in the composition of these pineapple genotypes. Sucrose was the dominant sugar in all 26 genotypes, while citric acid was the principal organic acid. Potassium, calcium and magnesium were the major mineral constituents. The ascorbic acid (AsA) content ranged from 5.08 to 33.57 mg/100 g fresh weight (FW), while the total phenolic (TP) content varied from 31.48 to 77.55 mg gallic acid equivalents (GAE)/100 g FW. The two parameters in the predominant cultivars Comte de Paris and Smooth Cayenne were relative low. However, MD-2 indicated the highest AsA and TP contents (33.57 mg/100 g and 77.55 mg GAE/100 g FM, respectively), and it also showed the strongest antioxidant capacity 22.85 and 17.30 μmol TE/g FW using DPPH and TEAC methods, respectively. The antioxidant capacity of pineapple was correlated with the contents of phenolics, flavonoids and AsA. The present study provided important information for the further application of those pineapple genotypes.

## 1. Introduction

Pineapple [*Ananas comosus* (L.) Merr.], is the third most important fruit crop in the tropical and subtropical regions of the world, only preceded by banana and citrus [1]. In 2011, the world production of pineapple fruit was 21,865,383 t and China is one of the major pineapple producers, accounting for 6.2% of the world’s pineapple production (1,351,367 t) [2]. In China, nearly 80% of the pineapple fruit is consumed as fresh fruit in the domestic market, and the remainder is processed to produce canned fruit and concentrated juice, which mainly go to the export market [3].

Pineapple is an important source of sugars, organic acids and some essential minerals for human nutrition and its quality of good flavor, aroma, juiciness and sweetness is well known and appreciated by consumers [4]. In addition, pineapple is also rich in health-promoting antioxidants, such as ascorbic acid, flavonoids, and other phenolic compounds related to antioxidant activities [5,6], which are important indexes for fruit acceptability to the consumer and processor. Several studies on the physico-chemical composition of some pineapple cultivars grown outside China have been reported [7,8,9,10,11]. However, the antioxidant activity, total phenolics and flavonoids of different pineapple cultivars are rarely documented [6]. Furthermore, no detailed research was carried out on the physico-chemical and nutritional properties of pineapple grown in China.

In China, the pineapple industry is dominated by the two cultivars of Comte de Paris and Smooth Cayenne, with the former occupying more than 80% of the planting areas. In order to breed new pineapple cultivars to solve the problem of variety degeneration caused by the use of single varieties, in 2010, the Ministry of Agriculture and the South Subtropical Crop Research Institute (Chinese Academy of Tropical Agricultural Sciences) jointly established a pineapple germplasm repository. Currently, the germplasm nursery conserves 130 pineapple genotypes of worldwide genetic variability. Therefore, the aim of the present study is to analyze and evaluate the physico-chemical, antioxidant properties and minerals of 26 pineapple genotypes grown in China. The results will provide basis for the selection of appropriate pineapple genotypes to breed new cultivars with improved nutritional quality, high content on bioactive compounds and suitable edible or processing quality.

## 2. Results and Discussion

### 2.1. Quality Properties

The quality properties of the 26 pineapple genotypes are presented in Table 1. Significant differences (*p* < 0.05) were detected in all measured parameters. Fruit weight ranged from 555.0 to 1564.5 g, with Giant Kew being the smallest and Sriracha the largest. Our results showed a similar fruit weight for Pattavia compared with those reported by Chuenboonngarm *et al.* [7], but a lower fruit weight for Smooth Cayenne as stated by Chen *et al.* [10]. Wide variations of TSS (total soluble solids) and TA (titratable acidity) (10.25 and 26.78% C.V. values, respectively) were found among the studied pineapple genotypes. TSS varied from 12.55 (Pearl) to 20.45 (Ripley), while the TA values ranged from 0.46 (Tainon 13) to 1.23% of citric acid (Smooth Cayenne #2), which was higher than previous reports for MD-2 and Smooth Cayenne cultivars [12]. Accordingly, these variations in TSS and TA led to great differences in TSS/TA ratio, which ranged from 17.15 in Smooth Cayenne #2 to 41.08 in Ripley. It is generally recognized that the TSS/TA ratio is the most reliable parameter index for evaluating pineapple fruit quality. To obtain high quality pineapple fruit, those cultivars with TSS/TA ratio from 20 to 40 were recommended by Soler [13]. In this study, 73.1% of the pineapple genotypes fell within the range. Therefore, it can be assumed that these genotypes had a better quality and acceptability. The pH values ranged from 3.58 (Smooth Cayenne #1) to 4.24 (Queensland Cayenne), which were in accordance with other data reported in literature [12]. Pineapple is regarded as a good source of ascorbic acid (AsA), which varies from 0.1 to 44 mg/100 g [14]. In this study, a wide range in the AsA contents was also observed. MD-2 had the highest content (33.57 mg/100 g) while Smooth Cayenne #1 had the lowest (5.08 mg/100 g). Similar findings have been published for pineapples of different cultivars [12]. In addition, C.V. values indicated that the AsA content was the most variable property (48.70%) while the least variable was pH (4.59%).

**Table 1:** Fruit weight, total soluble solids (TSS), titratable acidity (TA), TSS/TA ratio, pH, and ascorbic acid (AsA) of 26 pineapple genotypes from China.

Genotypes	Fruit Weight (g)	TSS (°Brix)	TA (% Citric Acid)	TSS/TA	pH	AsA (mg/100 g)
Comte de Paris	1061.2^de^	16.94^hi^	0.66^jk^	26.63^de^	3.93^bcdef^	10.07^hi^
CPM	1006.4^def^	16.38^jk^	0.91^d^	18.04^ij^	4.21^ab^	16.59^cd^
Fresh Premium	783.4^hij^	18.80^d^	0.59^m^	31.70^c^	4.04^abcd^	7.51^no^
Giant Kew	555.0^k^	17.70^fg^	1.12^b^	15.86^k^	4.15^abc^	12.39^of^
Kallara local	895.3^efgh^	17.40^gh^	0.72^gh^	24.18^fg^	4.09^abcd^	15.99^d^
MacGregor	1030.5^def^	20.11^b^	0.79^f^	25.44^ef^	4.14^abc^	13.63^e^
MD-2	1132.8^cd^	16.12^kl^	0.53^opq^	30.26^c^	4.13^abc^	33.57^a^
Nanglae	910.0^efgh^	16.20^jk^	0.68^hij^	24.21^fg^	3.95^abcde^	17.02^c^
New Puket	862.4^fghi^	16.30^jk^	0.54^nopq^	31.36^c^	4.10^abcd^	13.99^e^
Pattavia	1151.0^cd^	15.65^m^	0.85^e^	18.46^hij^	3.81^def^	9.10^jk^
Pearl	1290.3^bc^	12.55^o^	0.61^klm^	20.55^h^	3.61^f^	6.70^op^
Phetchaburi #2	1323.5^b^	16.93^hi^	0.71^ghi^	23.82^fg^	4.23^a^	21.57^b^
Puket	945.9^efgh^	16.43^jk^	0.52^pq^	31.72^c^	4.02^abcd^	8.72^jkl^
Queensland Cayenne	809.2^ghij^	17.80^efg^	0.69^ghij^	25.63^ed^	4.24^a^	8.21^klmn^
Ripley	705.1^ijk^	20.45^a^	0.51^q^	41.08^a^	3.91^abcde^	8.48^jklm^
Smooth Cayenne #1	1137.6^cd^	16.55^ijk^	0.73^g^	22.57^g^	3.58^f^	5.08^q^
Smooth Cayenne #2	1072.5^de^	14.45^n^	1.23^a^	11.75^l^	3.86^cdef^	7.68^mn^
Sriracha	1564.5^a^	16.15^kl^	0.81^ef^	19.90^hi^	3.85^cdef^	10.82^gh^
Tainon 6	1171.6^bcd^	16.70^ij^	0.61^klm^	27.23^de^	4.16^abc^	11.09^g^
Tainon 11	860.1^k^	18.12^ef^	0.68^ij^	26.89^de^	4.04^abcd^	14.38^e^
Tainon 13	1010.2^def^	15.70^lm^	0.46^r^	33.97^b^	3.90^bcdef^	10.62^gh^
Tainon 17	988.9^defg^	18.20^e^	0.57^mnop^	31.80^c^	3.99^abcd^	9.39^ij^
Tainon 18	1006.8^def^	17.34^gh^	0.62^kl^	28.06^d^	4.03^abcd^	7.99^lmn^
Tainon 19	1055.2^de^	19.40^c^	0.58^lmn^	34.52^b^	4.22^ab^	16.93^c^
Tainon 20	555.1^k^	14.40^n^	0.57^lmno^	25.46^ef^	3.69^ef^	6.15^p^
Tradsrithong	647.4^jk^	16.37^jk^	0.97^c^	17.03^jk^	4.06^abcd^	13.61^e^
Means	975.8	16.89	0.70	25.70	4.00	12.20
C.V. (%)	24.10	10.25	26.78	25.94	4.59	48.70

Note: Results expressed as means. Means in the same line with different letters are significantly different (*p* < 0.05).

### 2.2. Sugars and Organic Acids

The sugar profile plays an important role in the flavour characteristics and commercial assessment of pineapple fruit quality [15]. As shown in Table 2, sucrose was found to be the dominant sugar in all the genotypes, followed by glucose and fructose, and the results were consistent with previously findings [10,16]. Sucrose content varied from 45.22 (Pearl) to 89.46 mg/g FW (Puket).

**Table 2:** Sugar and organic acid contents (mg/g FW) of 26 pineapple genotypes from China.

Genotypes	Glucose	Fructose	Sucrose	Total Sugars	Citric	Malic	Quinic	Total Organ Acids
Comte de Paris	33.60^b^	31.41^a^	60.65^i^	125.66^c^	3.37^hij^	0.90^fg^	0.50^l^	5.09^e^
CPM	14.80^m^	13.21^p^	72.14^e^	100.15^jk^	4.21^de^	0.74^hi^	0.83^ij^	5.78^d^
Fresh Premium	34.90^a^	31.13^a^	63.85^gh^	129.88^b^	4.03^ef^	1.03^cde^	1.26^ab^	6.32^c^
Giant Kew	23.23^g^	22.65^g^	60.41^i^	106.32^i^	4.44^cd^	0.53^k^	0.93^h^	5.90^d^
Kallara local	23.21^g^	22.69^g^	61.97^ghi^	107.88^hi^	3.46^hi^	0.75^hi^	0.96^h^	5.16^e^
MacGregor	18.38^l^	16.71^m^	63.86^gh^	98.94^jkl^	3.03^jkl^	0.71^hi^	1.18^bcd^	4.93^ef^
MD-2	14.63^m^	13.06^p^	78.99^d^	106.67^i^	2.88^klm^	1.05^cd^	0.94^h^	4.88^efg^
Nanglae	27.13^c^	23.37^d^	81.80^c^	132.29^b^	2.62^m^	1.11^cd^	1.29^a^	5.15^e^
New Puket	21.61^i^	19.00^k^	83.81^bc^	124.41^cd^	4.63^bc^	1.05^cde^	1.22^abc^	6.90^b^
Pattavia	25.06^e^	23.81^c^	52.12^j^	100.99^j^	2.18^n^	0.98^def^	0.92^hi^	4.08^h^
Pearl	19.14^jk^	17.23^l^	45.22^k^	81.59^p^	2.63^m^	0.51^k^	0.52^l^	3.66^i^
Phetchaburi #2	25.69^d^	23.02^ef^	89.08^a^	137.80^a^	1.14^p^	0.30^l^	0.69^k^	2.13^k^
Puket	22.60^h^	20.24^j^	89.46^a^	132.30^b^	4.80^b^	1.05^cd^	1.23^abc^	7.08^b^
Queensland Cayenne	13.74^n^	13.08^p^	69.88^ef^	96.71^lm^	3.80^fg^	0.80^gh^	1.22^abc^	5.83^d^
Ripley	19.52^j^	15.99^n^	50.71^j^	86.22^o^	2.78^lm^	0.79^gh^	0.94^h^	4.51^g^
Smooth Cayenne #1	24.25^f^	21.03^h^	64.76^g^	110.05^gh^	4.44^cd^	1.61^a^	1.02^fgh^	7.07^b^
Smooth Cayenne #2	14.27^mn^	15.34^o^	69.32^ef^	98.93^jkl^	3.08^jkl^	0.94^ef^	1.01^fgh^	5.03^e^
Sriracha	21.31^i^	17.05^l^	52.29^j^	90.65^ n^	2.60^m^	0.81^gh^	1.13^cde^	4.54^fg^
Tainon 6	23.36^g^	21.00^hi^	68.89^f^	112.25^fg^	1.85^o^	0.59^jk^	1.08^efg^	4.05^h^
Tainon 11	18.84^kl^	17.14^l^	61.13^hi^	97.11^klm^	3.19^ijk^	0.92^f^	0.98^gh^	5.09^e^
Tainon 13	27.25^c^	22.73^fg^	51.86^j^	101.83^j^	1.41^p^	0.60^jk^	0.82^ij^	2.82^j^
Tainon 17	23.64^g^	20.71^i^	77.08^d^	121.44^e^	3.56^gh^	0.77^hi^	0.83^ij^	5.17^e^
Tainon 18	19.22^kl^	16.09^n^	78.79^d^	114.11^j^	1.97^no^	0.59^jk^	0.63^k^	3.13^j^
Tainon 19	26.23^d^	23.28^de^	46.34^k^	95.85^lm^	3.47^hi^	0.88^fg^	0.79^j^	5.14^e^
Tainon 20	27.55^c^	23.25^de^	71.16^ef^	121.96^de^	3.36^hij^	0.69^ij^	0.73^jk^	4.78^efg^
Tradsrithong	27.15^c^	25.35^b^	85.38^b^	137.89^a^	5.78^a^	1.21^b^	1.10^def^	8.09^a^
Means	22.70	20.37	67.34	110.38	3.26	0.84	0.95	5.09
C.V. (%)	23.85	23.88	19.42	14.60	33.52	31.47	23.48	26.68

Note: Results expressed as means. Means in the same line with different letters are significantly different (*p* < 0.05).

High values of sucrose content were also found in Phetchaburi #2 (89.08 mg/g FW), Tradsrithong (85.38 mg/g FW), New Puket (83.81 mg/g FW) and Nanglae (81.80 mg/g FW). Glucose was the second sugar of amount in pineapple and it varied from 13.74 to 34.90 mg/g FW in Queensland Cayenne and Fresh Premium, respectively. Fructose content demonstrated the lowest level among all the sugars measured in this study. It varied from 13.08 mg/g in Queensland Cayenne to 31.41 mg/g FW in Comte de Paris. The concentrations of total sugars as well as individual sugars were found statistically significant (*p* < 0.05) in Table 2. Total sugar contents in Tradsrithong (137.89 mg/g FW) and Phetchaburi #2 (137.80 mg/g FW) fruits were the highest, while the lowest was in Pearl (81.59 mg/g FW). Compared with persimmon, of which total sugar levels were between 106 and 178 mg/g FW [17], and apple with total sugar content of 115 to 183 mg/g FW [18], pineapple fruit had a lower total sugar content. But compared with elderberry, which contained 68.53–104.16 mg/g FW [19], pineapple fruit was rich in total sugars. In addition, we also found these genotypes had the similar contents of glucose and fructose, and the former was almost always higher than the latter except for in Smooth Cayenne #2. Similarly, glucose and fructose contents had even similar and greater variability than sucrose content when compared by their C.V. values (23.85% and 23.88% to 19.42%, respectively).

In all the pineapple genotypes, the major organic acids were malic, citric and quinic acids (Table 2). Statistically significant differences in the individual organic acids and total acid contents were detected among the genotypes. Citric acid was the predominant organic acid, which ranged from 1.14 to 5.78 mg/g FW (Phetchaburi #2, Tradsrithong). Quinic acid ranged from 0.50 to 1.29 mg/g FW (Comte de Paris, Nanglae) and malic acid ranged from 0.30 to 1.61 mg/g FW (Phetchaburi #2, Smooth Cayenne #1). In the study, the highest content of total organic acids was found in Tradsrithong (8.09 mg/g FW) and the lowest in Phetchaburi #2 (2.13 mg/g FW), which was lower than that in apple (6.00–14.00 mg/g FW) [18] and strawberry (5.0–11.2 mg/g FW) [20]. But compared with mango and elderberry, which contained organic acid from 0.59 to 1.79 mg/g FW [21], and 4.48 to 6.38 mg/g FW [19], respectively, pineapple genotypes had a much wider range of organic acid content. However, when we compared the compositions of organic acids, the results were similar to the two studies which both reported high levels of citric and low level of malic acids in pineapple [11,22]. In addition, Cáfimara *et al.* [23] demonstrated that pineapple fruit and juices contained trace levels of quinic, oxalic and succinic acid in addition to higher levels of citric and malic acids.

### 2.3. Total Phenolic and Flavonoid Contents and Antioxidant Activity

Phenolic such as flavonoids, phenolic acids, and other polyphenolic compounds are considered major contributors to the antioxidant activity of vegetables and fruits. We determined total phenolic and flavonoid contents of the 26 pineapple genotypes, and the detailed results are shown in Table 3. A significant variation in total phenolic content (TPC) was observed, which ranged from 31.48 to 77.55 mg gallic acid equivalents (GAE) /100g fresh weight (FW). The highest level of TPC was observed in MD-2 and the lowest in CPM. A recent study showed that the pulp of non-transformed pineapple extracted by ethanol and that of transformed pineapple extracted by distilled water both possessed higher TFC values (65 and 95 mg GAE/100g FW) [6]. In comparison with apple and spine grape, which contained TPC ranging from 105.4 to 269.7 mg GAE/100 g FW [24], and 157 to 365 mg GAE/100 g FW [25], respectively, pineapple fruit showed significantly lower TPC. But compare with avocado and pitaya, which contained TPC 21.86 and 27.52 mg GAE/100 g, respectively [26], pineapple fruit indicated significantly higher TPC.

The values of total flavonoid content (TFC) varied significantly from 6.16 mg rutin equivalents (RE)/100 g FM in Smooth Cayenne #1 to 34.50 mg RE/100 g FM in Comte de Paris (Table 3). The TFC in non-transformed and transformed pineapple pulp were 4.5 and 13.0 mg RE/100g FW, respectively [6] as previously reported. In comparison with spine grape, which contained TFC from 84 to 244 mg RE/100 g FM [25], pineapple fruit showed significantly lower TFC.

**Table 3:** Total phenolic contents (TPC), total flavonoid contents (TFC) and antioxidant activity determined by the DPPH and TEAC assays of 26 pineapple genotypes from China.

Genotypes	TPC (mg GAE/100 g FW)	TFC (mg RE/100 g FW)	DPPH (μmol TE/g FW)	TEAC (μmol TE/g FW)
Comte de Paris	48.01^jk^	34.50^a^	4.25^j^	5.71^mn^
CPM	31.48^n^	8.50^n^	3.68^j^	4.10^o^
Fresh Premium	56.21^efg^	17.24^g^	8.08^f^	9.28^f^
Giant Kew	41.94^l^	11.60^k^	3.79^j^	5.41^n^
Kallara local	53.01^gh^	6.19^o^	5.60^i^	7.04^jk^
MacGregor	55.15^fgh^	18.81^f^	13.55^c^	12.24^d^
MD-2	77.55^a^	27.31^b^	22.85^a^	17.30^a^
Nanglae	53.72^fgh^	11.67^k^	6.84^h^	6.85^k^
New Puket	53.61^fgh^	12.36^jk^	9.32^e^	7.80^i^
Pattavia	37.48^m^	10.31^l^	5.60^i^	7.23^j^
Pearl	56.84^ef^	19.64^e^	11.43^d^	10.72^e^
Phetchaburi #2	53.90^fgh^	10.53^l^	8.44^f^	7.63^i^
Puket	47.83^jk^	13.14^j^	5.44^i^	5.81^m^
Queensland Cayenne	70.69^b^	20.10^de^	14.76^b^	14.24^b^
Ripley	54.20^fgh^	26.07^c^	9.99^e^	13.11^c^
Smooth Cayenne #1	49.45^ij^	6.16^o^	7.20^gh^	6.55^l^
Smooth Cayenne #2	53.43^gh^	12.62^j^	5.18^i^	8.74^h^
Sriracha	47.71^jk^	7.97^n^	5.13^i^	5.65^mn^
Tainon 6	48.01^jk^	16.24^h^	7.04^h^	7.59^i^
Tainon 11	48.23^jk^	11.59^k^	9.88^e^	7.59^i^
Tainon 13	60.86^cd^	20.67^d^	11.69^d^	10.85^e^
Tainon 17	45.05^k^	16.55^gh^	7.826^fg^	8.68^h^
Tainon 18	58.77^de^	9.26^m^	6.44^h^	8.93^gh^
Tainon 19	62.43^c^	12.62^j^	11.03^d^	9.10^fg^
Tainon 20	55.19^fgh^	13.91^i^	5.12^i^	6.39^l^
Tradsrithong	51.89^hi^	10.48^l^	6.99^h^	6.49^l^
Means	52.79	14.85	12.20	8.35
CV%	17.63	45.82	48.70	50.07

Note: Results expressed as means. Means in the same line with different letters are significantly different (*p* < 0.05).

The antioxidant capacity of fruits and vegetables is an important indicator of health promoters, and many methods have been developed to evaluate this particular capacity [25]. In this study, to better evaluate pineapple antioxidant capacity *in vitro*, the DPPH and TEAC assays were used to determine this index. The differences of antioxidant capacity in both assays among the investigated genotypes were statistically significant (*p* < 0.05) (Table 4). MD-2 showed the best antioxidant capacity in both assays (22.85 μmol of TE/g FW for DPPH and 17.30 μmol TE/g FW for TEAC) while CPM showed the weakest (3.68 μmol TE/g FW for DPPH and 4.10 μmol TE/g FW for TEAC) in the two methods. This suggested that MD-2 had a strong antioxidant capacity compared with other pineapple genotypes, which was a potential value for further development and utilization. In apple, the value of antioxidant capacity achieved by TEAC assay ranged from 3.35 to 7.40 μmol TE/g FW [24], and in citrus, the value obtained through DPPH assay varied from 2.66 to 4.57 μmol TE/g FW [27], indicating pineapple fruit had relative high antioxidant capacity.

Many studies have demonstrated correlations between bioactive compounds and antioxidant activities in numerous fruits and vegetables. However, little information is known concerning these types of correlations in pineapple. In this study, a significant correlation was found between the DPPH assay and TPC (R = 0.802; *p* < 0.01), TFC (R = 0.477; *p* < 0.05) or AsA (R = 0.527; *p* < 0.01) according to the data from all the pineapple genotypes, and the R value was lower for AsA (0.527) compared with to phenolics (0.802) (Table 4). Meanwhile, the TEAC assay closely correlated with both TPC (R = 0.806; *p* < 0.01) and TFC (R = 0.570; *p* < 0.01), whereas no significant correlation between the TEAC assay and AsA (R = 0.302; *p* > 0.05) was found. The presence/absence of correlation between the two assays and AsA may be due to a diverse sensibility of DPPH and TEAC assays for such classes of hydrophilic antioxidants. In addition, a direct correlation between the two assays of antioxidant activity demonstrated high correlation coefficients (R = 0.912; *p* < 0.01). Some studies suggest this may be caused by chemistry similarity between the two assays since both methods are based on the electron transfer reaction [27].

**Table 4:** Correlation coefficients between antioxidant content and antioxidant activity.

	TPC	TFC	AsA	DPPH	TEAC
TPC	1				
TFC	0.410 *	1			
AsA	0.326 ^ns^	0.099 ^ns^	1		
DPPH	0.802 **	0.477 *	0.527 **	1	
TEAC	0.806 **	0.570 **	0.302 ^ns^	0.912 **	1

Note: ns: non-significant; *: level of significance (* *p* < 0.05, ** *p* < 0.01).

### 2.4. Mineral Contents

The contents of seven different nutrient elements are shown in Table 5. K, an essential mineral for controlling the salt balance in human tissues, was the most abundant element present in pineapple fruit. The richest source of K was Giant Kew (2602 mg/100 g) and the lowest was Ripley (975 mg/100 g). Mg was the second abundant element in pineapple, which required by many enzymes, especially the sugar and protein kinase families of enzymes that catalyzed ATP-dependent phosphorylation reactions. The highest Mg content was observed in Tainon 18 (117.1 mg/100 g) and the lowest value in New Puket (44.4 mg/100 g FW). Ca was the third preponderant mineral in pineapple, which could be classified as a source of calcium for adults and be helpful in lowering blood pressure. The Ca contents in the tested genetypes ranged from 5.4 (Tainon 17) to 126.2 mg/100 g (Tainon 20). Some trace elements (e.g., Fe, Zn, Mn and Cu) in plants are known to be very low. However, in terms of biological activity, they are strikingly strong [28]. As shown in Table 5, the mineral composition showed lower levels of Zn and Cu, and relatively higher values of Mn and Fe (Table 5), which ranged from 32.3 to 222.7 mg/kg and 9.9 to 175.4 mg/kg. Zn and Cu contents varied from 3.1 to 48.6 mg/kg and 0 to 12.0 mg/kg. Based on the C.V. values, the variation in Zn was the highest (46.76%), and the highest Zn content was found in Giant Kew (48.6 mg/kg). Compared with a previous study [29], the contents of Mg, Fe and Cu in Comte de Paris in this study were much higher, but the levels of K, Ca and Zn were much lower while Mn content was similar. In total, the mineral contents could be affected by genotypes, soil nutrient content, time of harvest and climates. It is noteworthy that minerals are important not only for human nutrition, but for plant nutrition as well.

**Table 5:** Minerals of 26 pineapple genotypes from China referred to the dry matter (DM) content.

Genotypes	K (mg/100g)	Ca (mg/100g)	Mg (mg/100g)	Fe (mg/kg)	Zn (mg/kg)	Mn (mg/kg)	Cu (mg/kg)
Comte de Paris	985^o^^p^	18.3^lmn^	52.5^n^	15.3^n^	3.1^lm^	75.3^j^	3.8^o^
CPM	1227^ijk^	40.9^g^	75.1^e^	27.8^m^	8.5^g^	77.5^j^	4.7^mn^
Fresh Premium	1067^n^	42.5^g^	47.5^pq^	146.8^b^	3.8^kl^	113.5^f^	7.6^hi^
Giant Kew	2602^a^	66.6^d^	90.7^d^	113.2^c^	48.6^a^	222.7^a^	4.4^no^
Kallara local	1165^lm^	53.3^f^	51.3^no^	9.9^o^	4.7^jk^	67.3^k^	1.8^p^
MacGregor	907^q^	79.0^c^	49.4^op^	15.2^n^	5.8^hi^	77.8^j^	5.4^lm^
MD-2	1261^hi^	95.4^b^	99.4^b^	30.5^m^	7.7^g^	74.0^j^	1.1^p^
Nanglae	1730^d^	54.9^f^	75.2^e^	42.7^l^	10.1^f^	73.9^j^	10.7^bc^
New Puket	1148^m^	15.6^no^	44.4^r^	62.5^hi^	12.2^e^	43.9^m^	9.3^ef^
Pattavia	1387^g^	23.8^j^	57.2^lm^	66.0^gh^	3.7^l^	100.6^g^	6.6^jk^
Pearl	1472^f^	22.3^jk^	60.8^jk^	58.2^ijk^	5.4^hij^	94.7	9.8^de^
Phetchaburi #2	1294^h^	18.2^lmn^	67.8^g^	60.8^hij^	6.1^h^	32.3^n^	11.4^ab^
Puket	1192^klm^	17.1^mn^	55.4^m^	72.8^ef^	5.3^hij^	41.2^m^	9.8^de^
Queensland Cayenne	1209^jkl^	20.7^kl^	60.4^jk^	54.5^k^	2.3^m^	87.3^i^	0.0^q^
Ripley	975^p^	30.8^i^	46.4^qr^	38.5^l^	8.2^g^	137.7^d^	4.3^no^
Smooth Cayenne #1	1062^n^	35.9^h^	60.1^jk^	56.3^jk^	21.2^c^	83.9^i^	8.8^fg^
Smooth Cayenne #2	1224^ijk^	22.7^jk^	65.6^gh^	77.0^e^	3.4^l^	75.9^j^	6.3^jk^
Sriracha	1224^ijk^	20.3^kl^	59.1^kl^	70.8^fg^	3.5^l^	75.5^j^	12.0^a^
Tainon 6	2021^b^	62.9^e^	76.3^e^	175.4^a^	29.1^b^	150.6^c^	5.7^kl^
Tainon 11	990^op^	55.7^f^	71.1^f^	26.5^m^	5.2^hij^	95.5^h^	0.0^q^
Tainon 13	1844^c^	61.1^e^	64.4^hi^	28.9^m^	8.5^g^	92.8^h^	7.1^ij^
Tainon 17	1639^e^	5.4^p^	62.5^ij^	29.6^m^	4.8^ij^	161.6^b^	8.1^gh^
Tainon 18	1030^no^	42.2^g^	117.1^a^	88.5^d^	6.2^h^	84.3^g^	9.7^de^
Tainon 19	1391^g^	19.8^klm^	52.9^n^	61.3^hij^	4.7^ijk^	49.0^l^	10.4^cd^
Tainon 20	1241^ijk^	126.2^a^	93.7^c^	56.5^jk^	17.8^d^	131.3^e^	11.0^bc^
Tradsrithong	1250^hij^	14.0^o^	58.4^kl^	63.3^hi^	5.3^hij^	40.8^lm^	9.6^de^
Means	1328	41.0	66.0	59.6	9.4	90.8	6.9
CV%	28.41	69.72	26.87	64.90	106.89	46.76	51.81

Note: Results expressed as means. Means in the same line with different letters are significantly different (*p* < 0.05).

### 2.5. Cluster Analysis

**Figure 1:**
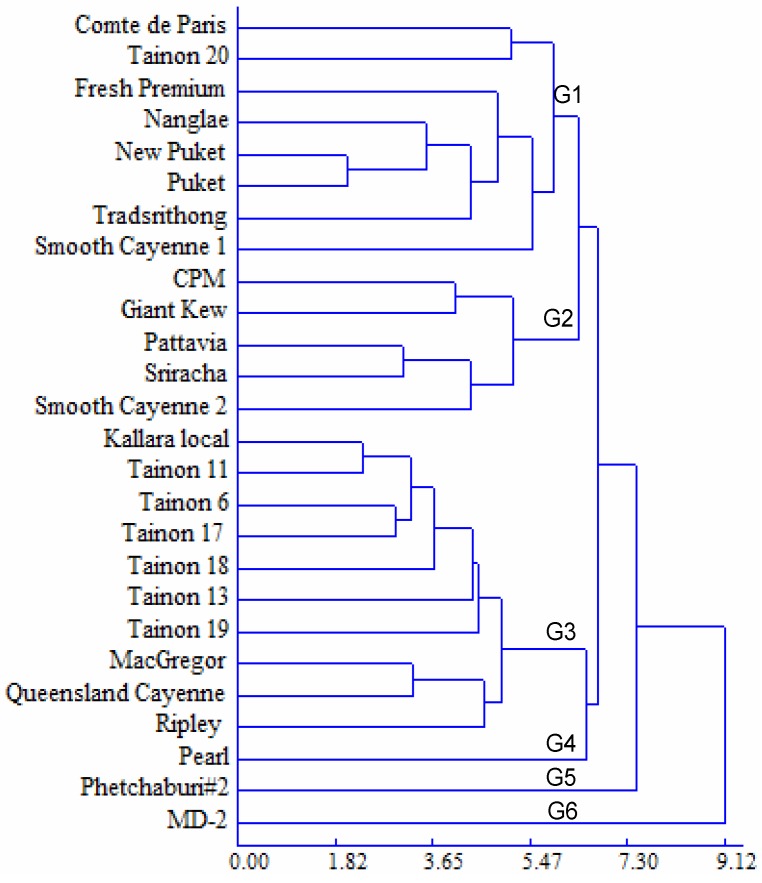
Dendrogram of 26 pineapple genotypes based on their physico-chemical composition and antioxidant properties.

Hierarchical cluster analysis was used to sort the pineapple genotypes based on the physico-chemical and antioxidant characteristics presented in Table 1, Table 2 and Table 3 (Figure 1). The 26 genotypes were clustered into six major groups. Group 1 (G1) was characterised by high total sugars, moderate TPC and low antioxidant capacity. Group 2 (G2) was associated with high TA values and low TSS/TA ratio, total sugars, TPC and antioxidant capacity. Ten genotypes belonged to group 3 (G3) with high TSS/TA ratio, TPC and antioxidant capacity. Pearl belonged to group 4 (G4) with the lowest TSS, sucrose content and total sugars, while Phetchaburi #2 belonged to group 5 (G5) with the second highest sucrose content and total sugars and the lowest organic acids. In addition, group 6 (G6) also had one genotype: MD-2, characterised by the highest AsA content, TPC and antioxidant capacity.

## 3. Experimental

### 3.1. Chemicals

Glucose, fructose, sucrose, citric acid, malic acid, quinic acid, gallic acid, rutin, 2,6-dichlorophenol-indophenol, Folin-Ciocalteu reagents, 2,2-diphenyl-1-picrylhydrazyl (DPPH), 2, 2-azinobis-(3-ethyl-benzothiazoline-6-sulphonic acid)-diammonium salt (ABTS) and 6-hydroxy-2,5,7,8-tetramethyl-chroman-2-carboxylic acid (Trolox) were purchased from Sigma-Aldrich Chemical Co. (Shanghai, China). The mineral standards were obtained from Sherwood Scientific Ltd. (Cambridge, UK). All the other chemicals were of analytical grade, and the solvents used for chromatography were HPLC grade (Tjshield, Tianjin, China).

### 3.2. Plant Materials

Twenty six pineapple genotypes were harvested at commercial maturity in 2012 from the germplasm repository, which located at South Subtropical Crop Research Institute in the Province of Guangdong, China (110°18′14.55″ E, 21°10′7.62″ N, and altitude 120.90 m). All the genotypes were grown under the same geographical conditions and with the same standard cultural practices. For each genotype, three replicates (consisting of five fruits each) were carried out (n = 3) and used for measurement and analysis.

### 3.3. Fruit Morphological and Quality Parameters

Fruits were weighed individually using a Mettler digital balance (±0.01 g). After that, the pulp was manually separated from the fruit and cut into small pieces to obtain homogeneous samples. Fruit pulp (200 g) was homogenized separately in a blender, and total soluble solids (TSS), pH, titratable acidity (TA) and ascorbic acid (AsA) contents were determined. TSS, expressed as Brix, was measured using a hand-held refractometer (ATC-20E, Atago, Tokyo, Japan). The pH values were measured with a digital pH metre (DL 25, Mettler Toledo, Greifensee, Switzerland). TA and AsA contents were determined according to the methods previously reported [30]. TA was expressed as % of citric acid. On the basis of the measured data, the TSS/TA ratio was calculated. The remaining flesh samples were immediately frozen and ground in liquid nitrogen and stored at −80 °C until use.

### 3.4. Extraction and Determination of Sugars and Organic Acids

Pineapple fruit samples were determined for the contents of individual sugars (glucose, fructose and sucrose) and organic acids (malic, citric and quinic acid). Homogenized samples (5 g) were immersed in aqueous ethanol (25 mL, 80%, v/v) for 30 min at 30 °C, the extracted samples were centrifuged at 10,000 *g* for 5 min (Eppendorf Centrifuge 5810R, Hamburg, Germany). The supernatant was made up to 25 mL with the same solvent and evaporated to dryness on 70 °C water bath. The residue was dissolved with 10 mL of twice distilled water and filtered before analysis.

Sugars and organic acids were all analysed using high-performance liquid chromatography (HPLC) (LC-20A, Shimadzu Corp., Kyoto, Japan). Analysis of sugars was carried out using an amino column (250 mm × 4.6 mm; Kromasil, Bohus, Sweden) with a flow rate of 1.0 mL/min and with column temperature maintained at 35 °C. For the mobile phase, acetonitrile and twice distilled water (70:30 v/v) solution was used, and a refractive index detector for identification. Organic acids were analysed in the Waters Atlantis T3 column (250 mm × 4.6 mm; Waters, Milford, MA, USA), and a UV detector set at 210 nm with a flow rate of 1.0 mL/min. For the mobile phase, 10 mM diammonium phosphate (adjusted to pH 2.70 with 1.0 M H_3_PO_4_) was used. For each analysis, 20μL of extract was used. Sugars and organic acids were identified and calculated with the help of corresponding external standards.

### 3.5. Sample Extraction for Determination of Total Phenolics, Flavonoids and Antioxidant Activity

Samples (1 g) were extracted with ultrasonic assistance in 10 mL of 80% methanol at 25 °C for 30 min in an external water bath. The extracted samples were centrifuged at 10,000 g for 20 min at 4 °C. The extraction procedure was repeated three times under identical conditions, and the combined supernatants were used to determine total phenolics, flavonoids and antioxidant activity.

### 3.6. Determination of Total Phenolic and Flavonoid Contents

Total phenolic content (TPC) was determined according to a previously described procedure [31]. Briefly, 0.5 mL of extract was mixed with 0.5 mL of Folin-Ciocalteu reagent (previously diluted 10-fold with distilled water) and allowed to stand at room temperature for 5 min, 1.0 mL of 7% sodium carbonate solution was added, and the mixture was placed at 37 °C in a water bath for 30 min. The absorbance was measured at 760 nm by a UV-visible spectrophotometer (UV-1700, Shimadzu Corp.). Quantification was done on the basis of a standard curve of gallic acid. Results were expressed as mg gallic acid equivalents (GAE)/100g fresh weight (FW). Total flavonoid content (TFC) was determined using the method of Jia *et al.* [32]. Briefly, 0.5 mL of extract was mixed with 2.25 mL of methanol solution in a test tube followed by addition of 0.15 mL of 5% NaNO_2_. After 5 min, 0.3 mL of 10% AlCl_3_ was added and finally 1.0 mL of 1 M NaOH was added after 6 min. The mixture was mixed well by vortexing, and the absorbance was measured at 510 nm. Results were expressed as mg rutin equivalents (RE)/100g FW.

### 3.7. Determination of Antioxidant Activity

#### 3.7.1. DPPH Free Radical Scavenging Capacity

DPPH free radical scavenging capacity was determined according to a modified method described by Bao *et al.* [31]. Briefly, 0.1 mL of the extract was added to 3.9 mL of a 0.06 mM DPPH solution stirred well and kept in the dark for 30 min. The absorption was measured at 517 nm. The antioxidant activity was expressed as μmol Trolox equivalents (TE)/g FW.

#### 3.7.2. Trolox Equivalent Antioxidant Capacity (TEAC)

The trolox equivalent antioxidant capacity (TEAC) was measured using the ABTS^+^ decoloration method [33]. ABTS was dissolved in water to a 7 mM concentration. The ABTS radical cation (ABTS^+^) was produced by reacting ABTS stock solution with 2.45 mM potassium persulfate and stand in the dark at room temperature for 16 h before use. 0.1 mL of the extract was mixed with 3.9 mL of diluted ABTS^+ ^ solution (Abs_734 nm_ = 0.700 ± 0.020). After 10 min the Abs_734 nm_ was measured and the antioxidant activity was expressed as μmol TE/g FW.

### 3.8. Mineral Contents

The mineral content was determined in dry ash samples at 550 °C and dissolved in HCl according to AOAC [34]. Calcium (Ca), magnesium (Mg), iron (Fe), zinc (Zn), manganese (Mn) and copper (Cu) contents were determined using inductively coupled plasma atomic emission spectrometer (ICP-AES) (Prodigy, Leeman Labs, Hudson, NH, USA). Potassium (K) contents were determined by using a flame photometer (M410, Sherwood Scientific Ltd., Cambridge, UK) with an air-propane flame.

### 3.9. Statistical Analysis

Statistical analyses were performed using SPSS version 11.5 software (SPSS Inc., Chicago, IL, USA). All data were collected and analysed by one-way analysis of variance (ANOVA). Significant differences among means at *p* < 0.05 were determined by Duncan’s multiple range tests. To evaluate variation between genotypes, coefficient of variations (C.V.) were calculated dividing relevant standard deviations by means and expressed as percentages. Hierarchical cluster analysis was used to group pineapple genotypes.

## 4. Conclusions

In this study, we determined the fruit physio-chemical properties, antioxidant activity and mineral contents of 26 pineapple genotypes grown in China. Our results showed statistically significant differences between genotypes on all determined parameters. Quantitatively, the major sugar and organic acid were found as sucrose and citric acid in pineapple, respectively. The highest content of total sugars was found in Tradsrithong and Phetchaburi#2, while the lowest was in Pearl. In terms of organic acids, the highest content of organic acids was found in Tradsrithong and the lowest in Phetchaburi #2. Moreover, we analysed the levels of antioxidants relevant to human health, such as ascorbic acid, phenolics and flavonoids, and evaluated the antioxidant activities using the DPPH and TEAC assays. We found that MD-2 had the highest AsA content, TPC and the strongest antioxidant capacity, while CPM showed the lowest TPC and the weakest antioxidant activity.

Amongst the 26 pineapple genotypes studied, the best for direct consumption was MD-2 for its appropriate TSS/TA ratio, high levels of bioactive compounds, higher fruit weight and minerals. In addition, the results also provided important information of pineapple genotypes which could be useful for fruit processing industry and selection of superior desirable genotypes for commercial cultivation. However, the pineapple quality is affected by many factors, such as region specialty, climate, soil characteristics and cultivation techniques, *etc.* Therefore, further studies on those related parameters are needed for the selection of excellent pineapple genotypes for fresh consumption, processing or breeding research.
